# Supplementary material for: Complex motion of steerable vesicular robots filled with active colloidal rods
Source: Sci Rep. 2023 Dec 20;13:22773. doi: 10.1038/s41598-023-49314-8 (PMC10733302; doi:10.1038/s41598-023-49314-8)
Supplement: Supplementary file 1 — Supplementary Information 1. [file 41598_2023_49314_MOESM1_ESM.pdf]

Two dimensional probability density map of finding a rod in the vesicle

We use kernel density estimation (KDE) with a Gaussian kernel to estimate the probability distribution of rods' relative location in the vesicle in the steady state. The probability to find a rod is measured per rectangular bin located at (w_i, h_j), where $i, j \in [1, 100]$. The bins are equally spaced in the rectangular region that spans between the lower and upper limit of the width of the vesicle along the w direction and of the height of the vesicle along the h direction.

Shape of the vesicle with respect to bending stiffness

The vesicle shape in steady state varies with vesicle stiffness. The vesicle stiffness is set by the harmonic angle "spring constant", κ_a, applied to every neighboring triplet of disks that composes the vesicle. The shape elongation is determined by eigenvalues and eigenvectors of the vesicle boundary gyration tensor. In two dimensions, the x-y component of the gyration tensor is

$s_{xy} = \frac{1}{2N^2} \sum_{i=1}^{N} \sum_{j=1}^{N} (x_i - x_j)(y_i - y_j)$

The square roots of the eigenvalues ($\sqrt{L1}, \sqrt{L2}$) are the characteristic principal-axis lengths (radii) of the ellipsoid that describesthe shape of the vesicle. The metric for the shape descriptor is defined as a ratio of the shortest gyration moment to the longest gyration moment: $Ratio(L1, L2) = \frac{\sqrt{L1}}{\sqrt{L2}}$. The ratio is plotted in Fig. S1 for each rod aspect ratio ($\alpha = 2, 3$) with various $\kappa_a = 50, 125, 250, 500, 1000 k_b T$, $\theta_{kink} = \pi/2, 5\pi/6$, and number densities $\rho_{N_a} = 0.1, 0.15, 0.2$. The value of the shape descriptor decreases with decreasing vesicle stiffness, which means that the vesicle is more elongated with more flexible boundaries. The reference points marked with black squares are from the vesicles containing no propelling rods. The shape descriptor deviates more from the reference point as vesicle stiffness decreases. The higher number density ρ_{N_a} leads to more elongation.

Vesicle shape with rounded tip

To showcase the robustness of our model, we also tested vesicular robots with rounded tips instead of discontinuous kinks (see Fig.S2). This model is inspired by experiments by Xin *et al.*[1], who created vesicles with non-uniform curvature by inserting two solid regions into a fluid membrane. In our simulations we realize the solid domains of a vesicle with perimeter 100σ and default bending rigidity $\kappa_a = 10, 100 k_B T$ (see Eq.(6) in main document) by imposing two regions of high bending rigidity ($\kappa_a = 1000 k_B T$). Depending on the relative bending rigidities, lengths (we used 23σ for each solid domain) and distances between the solid domains (3σ), the vesicular robots in this set-up also exhibit a pointy waterdrop shape with high curvature between the two solid domains. However, instead of a 'kink' with discontinuous curvature, the tip is continuously rounded. Vesicles which form a more rounded waterdrop ($\kappa_a = 100 k_B T$ for fluid membrane) mostly move linearly with parallel active rod stackings and can be considered equivalent to kinked vesicles with highly obtuse kink angles (see Fig. S2 (a)). Similarly, vesicles with a narrower tip region ($\kappa_a = 10 k_B T$ for fluid membrane) show circular trajectories with angled stacking of the active rods like in the single-kink model with highly acute kink angles (see Fig. S2 (b)). However, we have better control over the consistent directional motions in the single-kinked vesicle model, because integrating two solid domains introduces not only one region of high curvature in between, but also two additional highly curved regions at the other ends of the solid domains. Here the cluster formation of rods is also promoted leading to counteracting motions.

Supplementary videos

video1.mp4: **Forward linear motion** Trajectory of a linearly forward moving kinked vesicle with kink angle $\theta_{kink} = 5\pi/6$ containing two groups of active rods acting as actuators (aspect ratio $\alpha = 3$) with different onsets of activity. One group (yellow) is activated before the other group (navy) to control the speed of the vesicular superstructure. The inset shows a close-up view of the vesicle.

video2.mp4: **Backward linear motion** Trajectory of a linearly backward moving kinked vesicle with kink angle $\theta_{kink} = 5\pi/6$ containing two groups of active rods acting as actuators (aspect ratio $\alpha = 2$) with different onsets of activity. One group (yellow) is activated before the other group (navy) to control the speed of the vesicular superstructure. The inset shows a close-up view of the vesicle.

video3.mp4: **Transition from circular to linear motion** Trajectory of a forward moving kinked vesicle with varying kink angle θ_{kink} from $\pi/2$ to $5\pi/6$ containing active rods acting as actuators (aspect ratio $\alpha = 3$). The inset shows a close-up view of the vesicle.

video4: **Transition from linear to circular motion** Trajectory of a forward moving kinked vesicle with varying kink angle θ_{kink} from $5\pi/6$ to $\pi/2$ containing active rods acting as actuators (aspect ratio $\alpha = 3$). The inset shows a close-up view of the

vesicle.

video5: **Transition from linear to circular motion with two diffent velocities** Trajectory of a forward moving kinked vesicle with varying kink angle θ_{kink} from $5\pi/6$ to $\pi/2$ containing two groups of active rods acting as actuators (aspect ratio $\alpha = 3$) with different onsets of activity. The inset shows a close-up view of the vesicle.

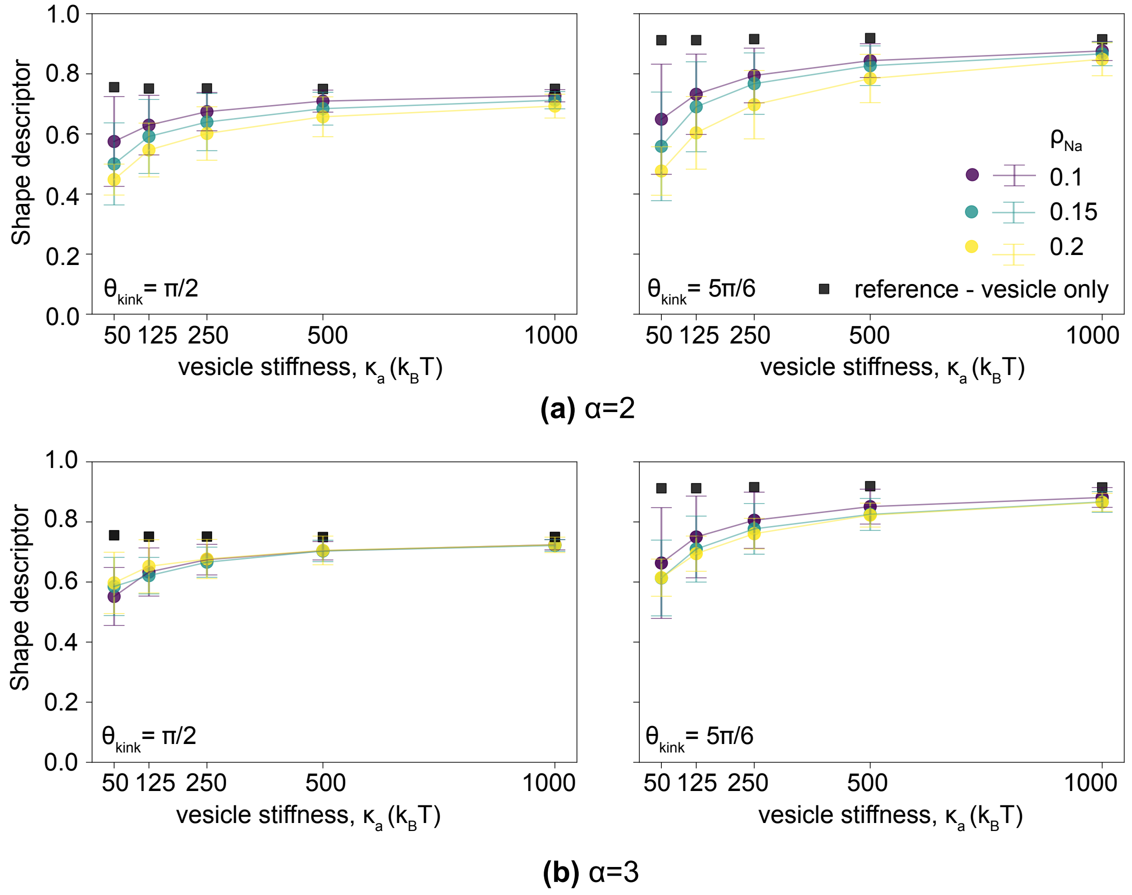

Figure S1. Shape elongation of the vesicle. Shape descriptor measure of the extent of elongation for different values of vesicle bending stiffness κ_a and different aspect ratios α of the rod. Color refers to the number densities $\rho_{N_a} = 0.1, 0.15, 0.2$. The marker is the averaged descriptor value with error bar of matching color. The reference system for each value of bending stiffness is from a vesicle with no active particles.

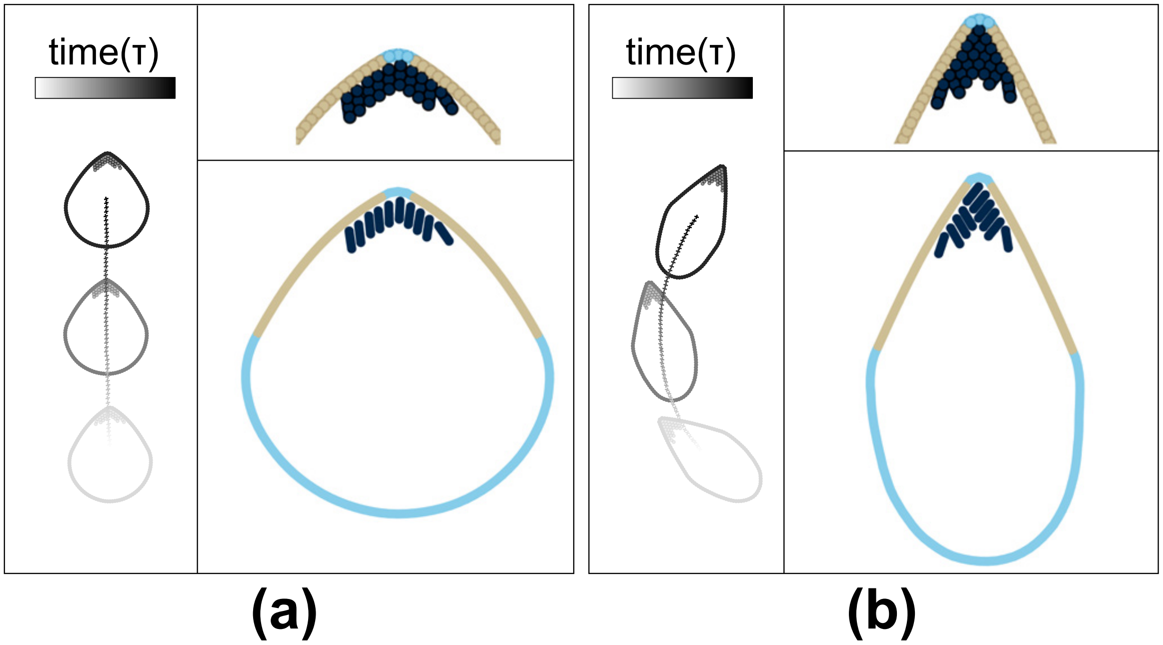

Figure S2. **Active rods confined in a continuously curved vesicle.** Sketch of vesicles with perimeter 100σ and continuous curvature containing rigid active rods (dark blue) with aspect ratio $\alpha = 3$. The rods (a) align parallel to the long axis of the vesicle at a wide tip, and (b) aligning at an angle with one side of the vesicle wall at a narrow tip. The beige color in the vesicle indicates the solid-like rigid section (23σ for each section) with $\kappa_a = 1000 k_B T$, and the blue color indicates the fluid-like section with (a) $\kappa_a = 100 k_B T$ and (b) $\kappa_a = 10 k_B T$. The distance in fluid domain between two solid domains is 3σ. The trajectories of each vesicle structure are described in the left column of each subfigure indicating (a) linear forward and (b) linear circular motion.

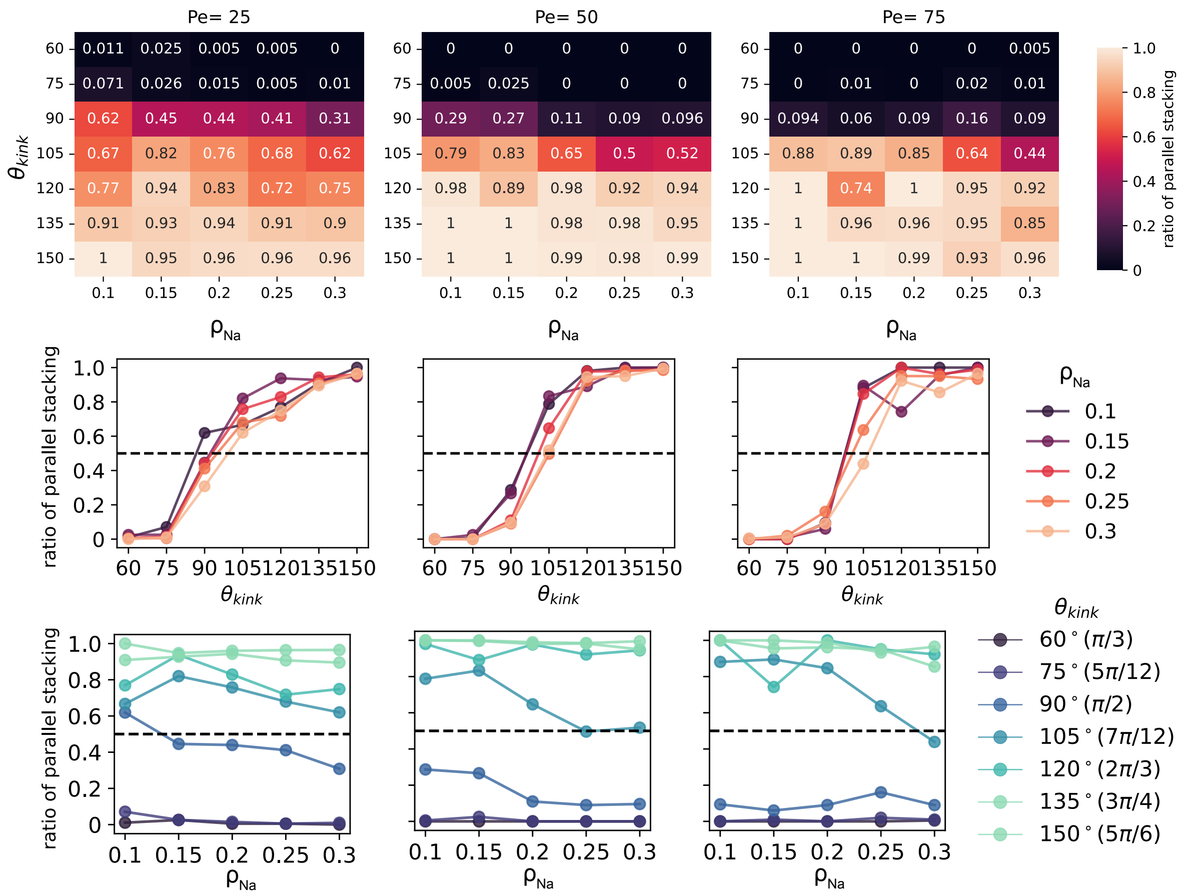

Figure S3. **Ratio of parallel vs. angled stacking plotted over vesicles with $\alpha = 2$ for each data point with corresponding Pe, θ_{kink} and ρ_{N_a}.** For each moving vesicle the stacking was measured either *parallel* or *angled*. Angled stacking corresponds to cases when the closest active rod particle to the kink aligns with the vesicle boundary; otherwise the rods are considered to be stacked in parallel.

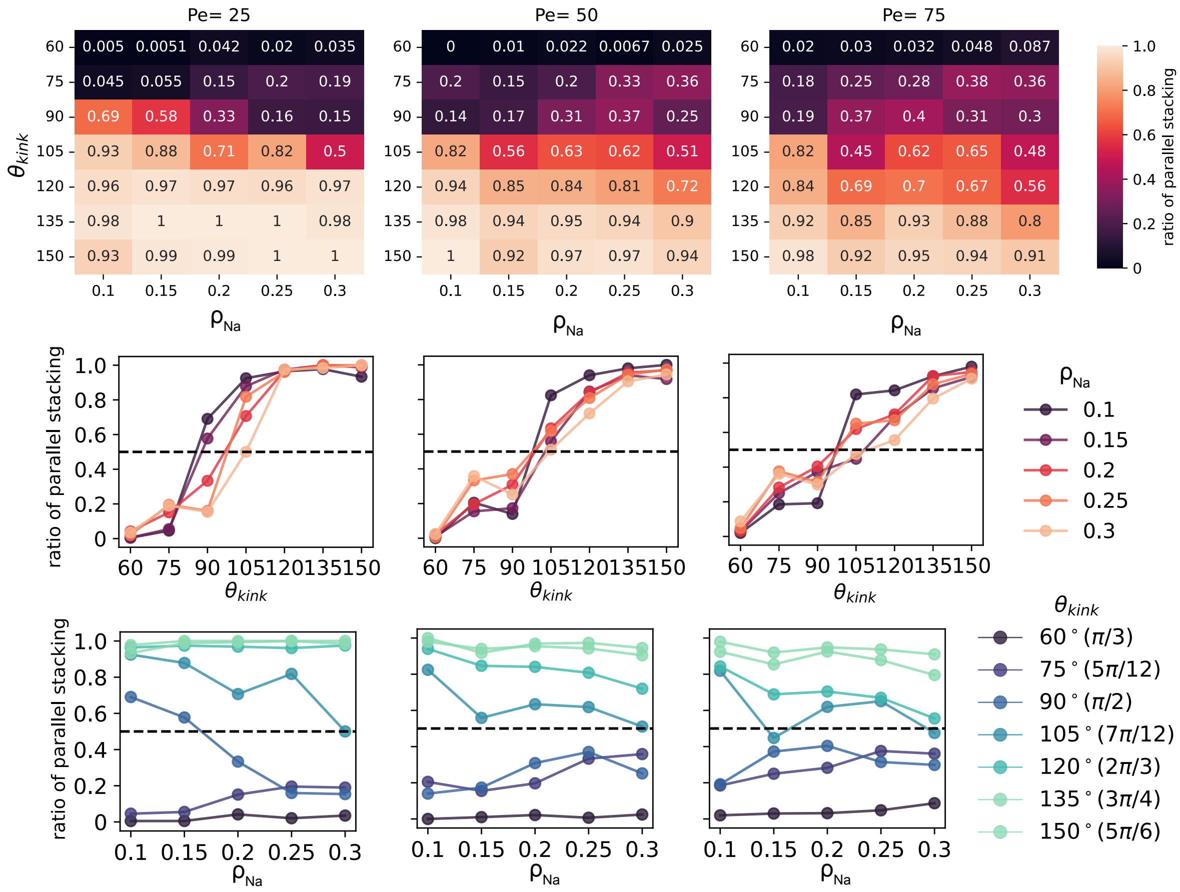

Figure S4. Ratio of parallel vs. angled stacking plotted for vesicles with $\alpha = 3$ for each data point with corresponding Pe, θ_{kink} and ρ_{N_a}. The measurement method is identical to that in Fig. S2.

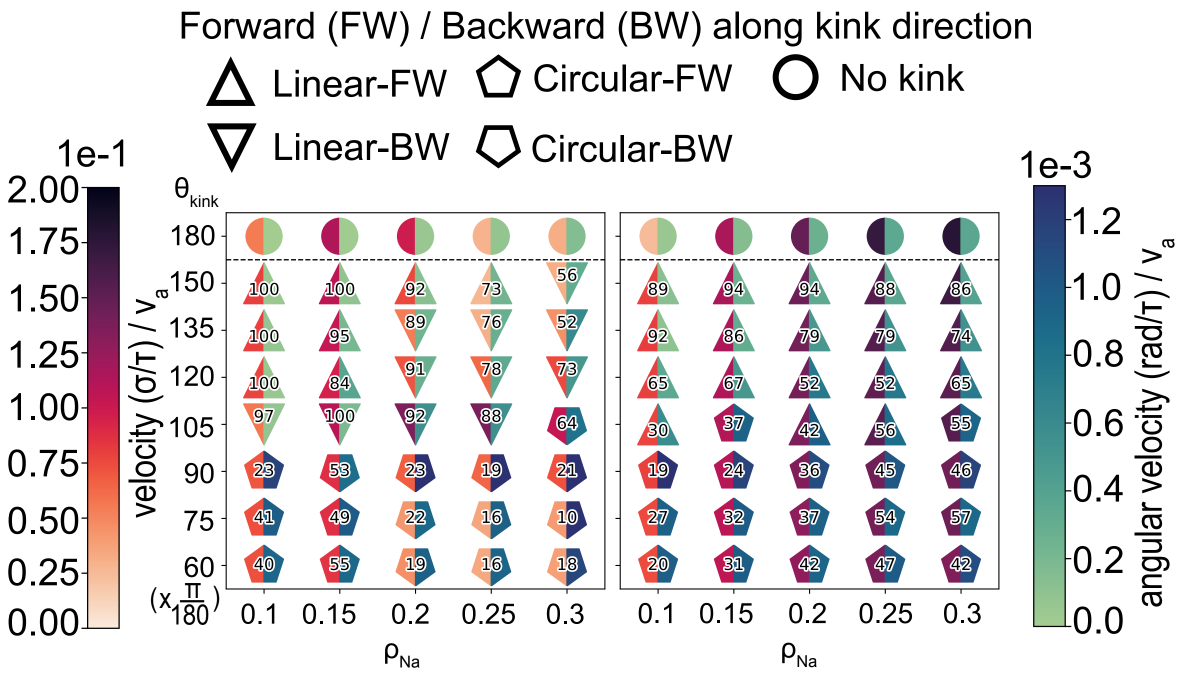

Figure S5. Chart of directional motion of the vesicle with regards to Pe=75, θ_{kink} and ρ_{N_a}. The color scheme is the same as in Fig. 1

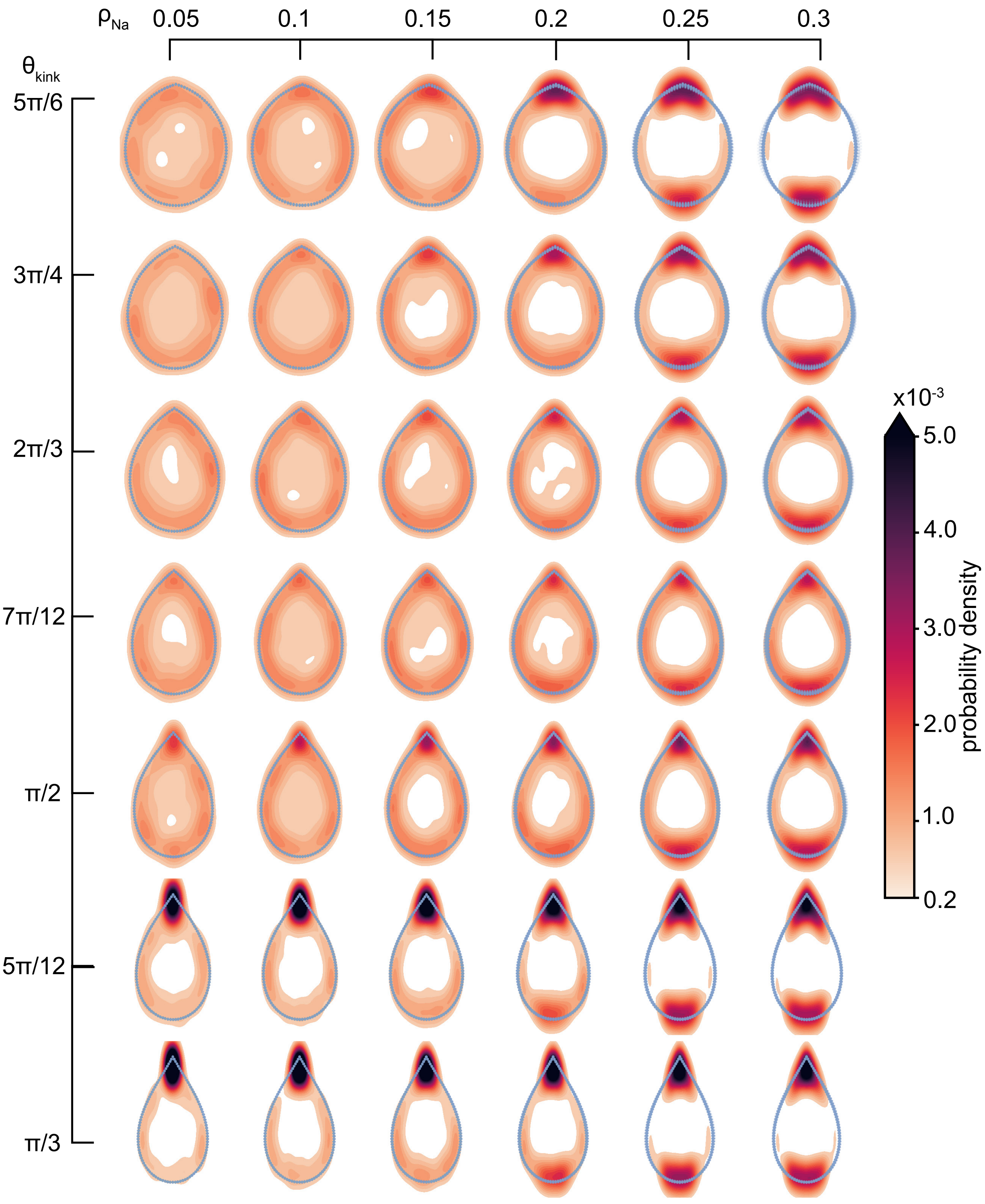

Figure S6. Probability density plot of enclosed particle $\alpha = 2$**,** $Pe = 25$**,** $\kappa_a = 1000k_BT$**.** The color scheme is the same as in Fig. 2

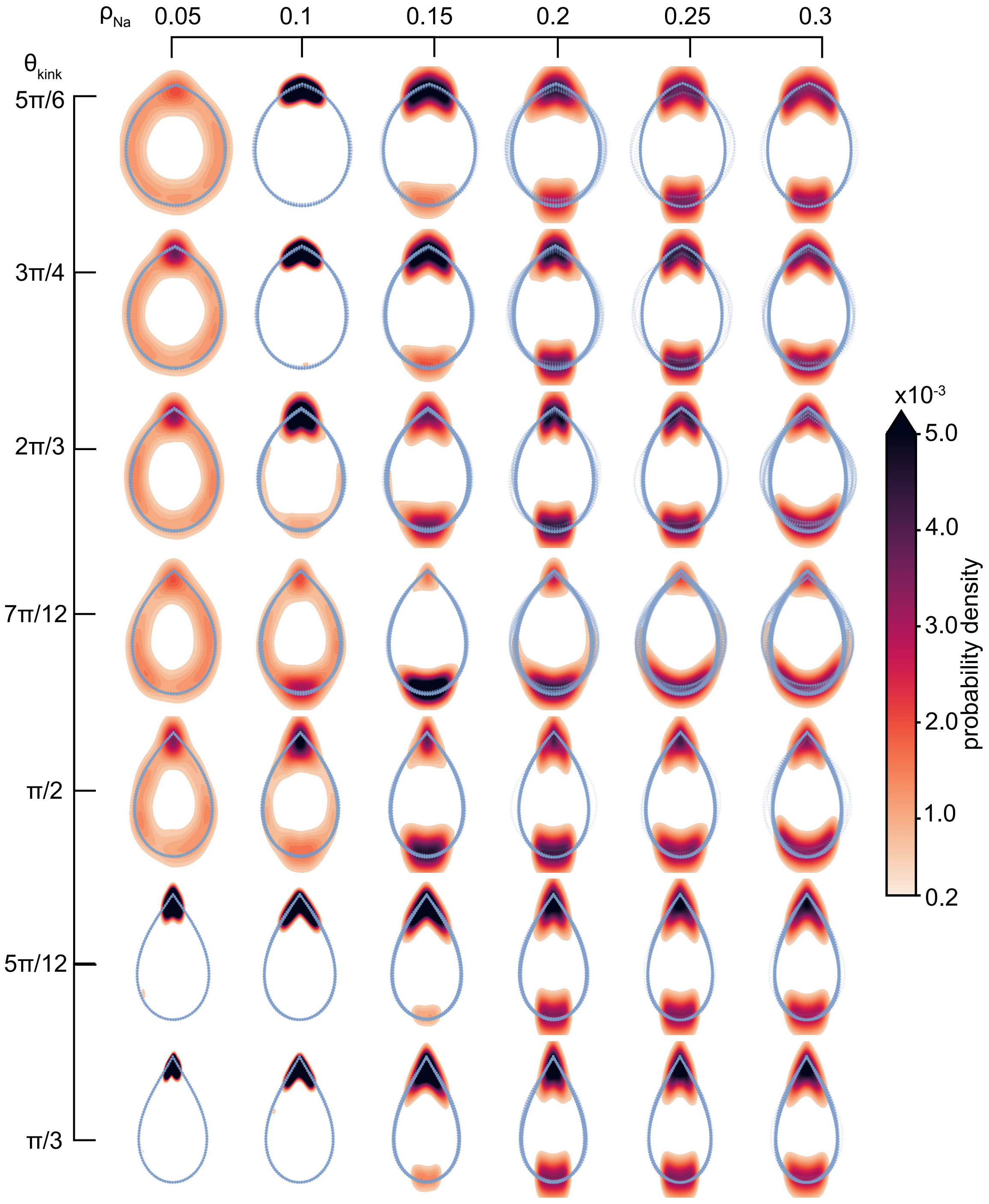

Figure S7. Probability density plot of enclosed particle $\alpha = 2$, $Pe = 50$, $\kappa_a = 1000k_BT$. The color scheme is the same as in Fig. 2

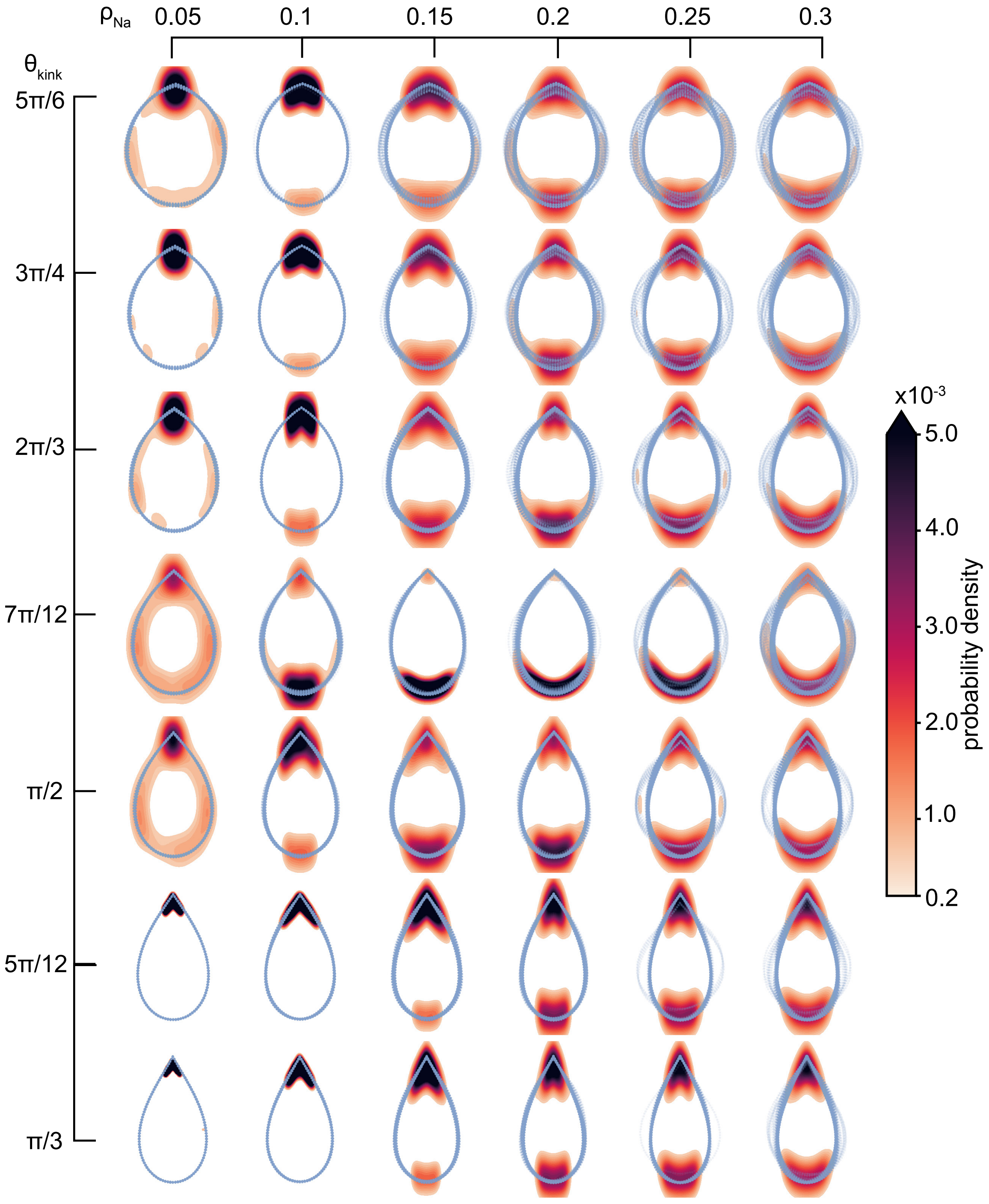

Figure S8. Probability density plot of enclosed particle $\alpha = 2$, $Pe = 75$, $\kappa_a = 1000 k_B T$. The color scheme is the same as in Fig. 2

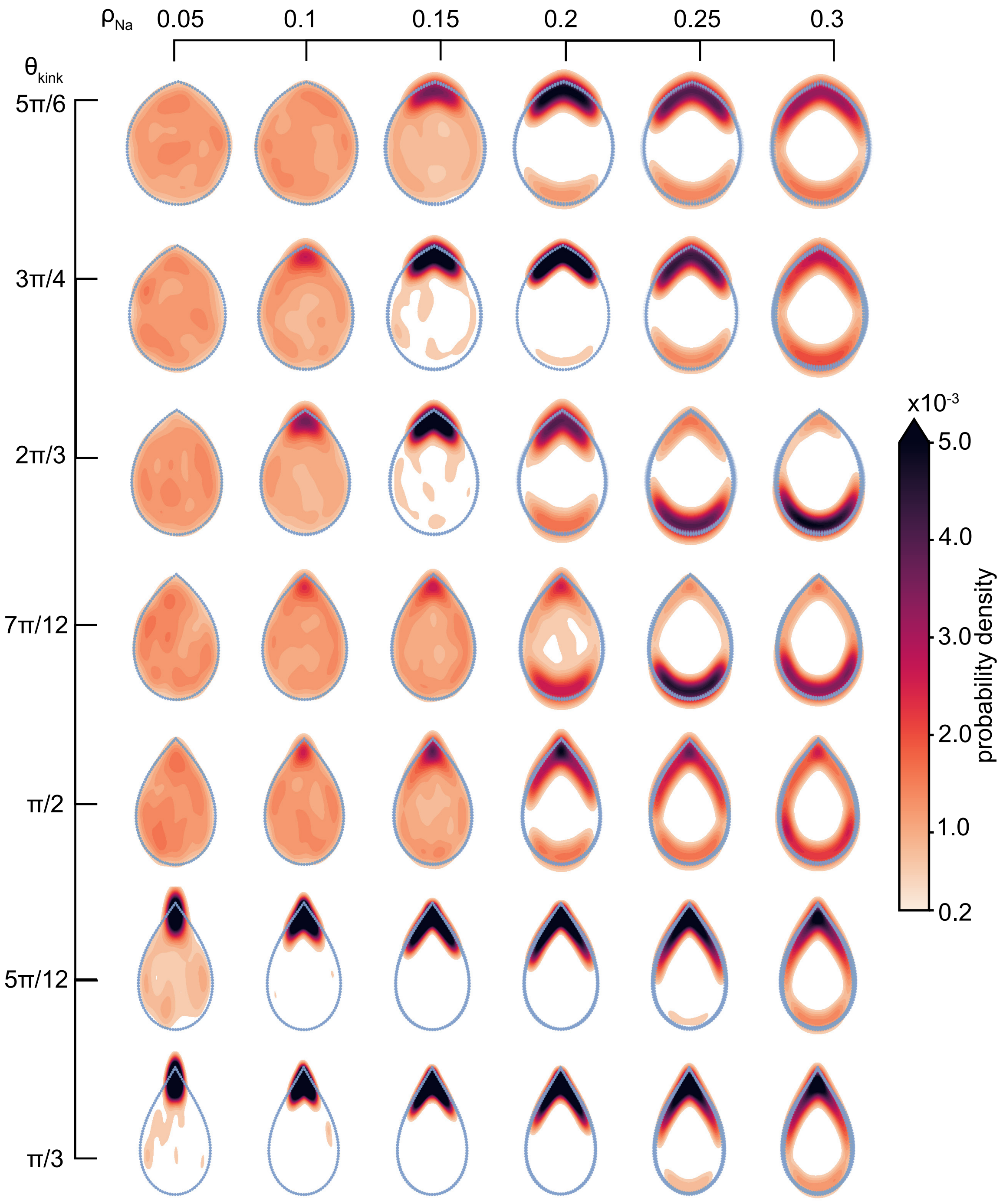

Figure S9. Probability density plot of enclosed particle $\alpha = 3$, $Pe = 25$, $\kappa_a = 1000k_BT$. The color scheme is the same as in Fig. 2

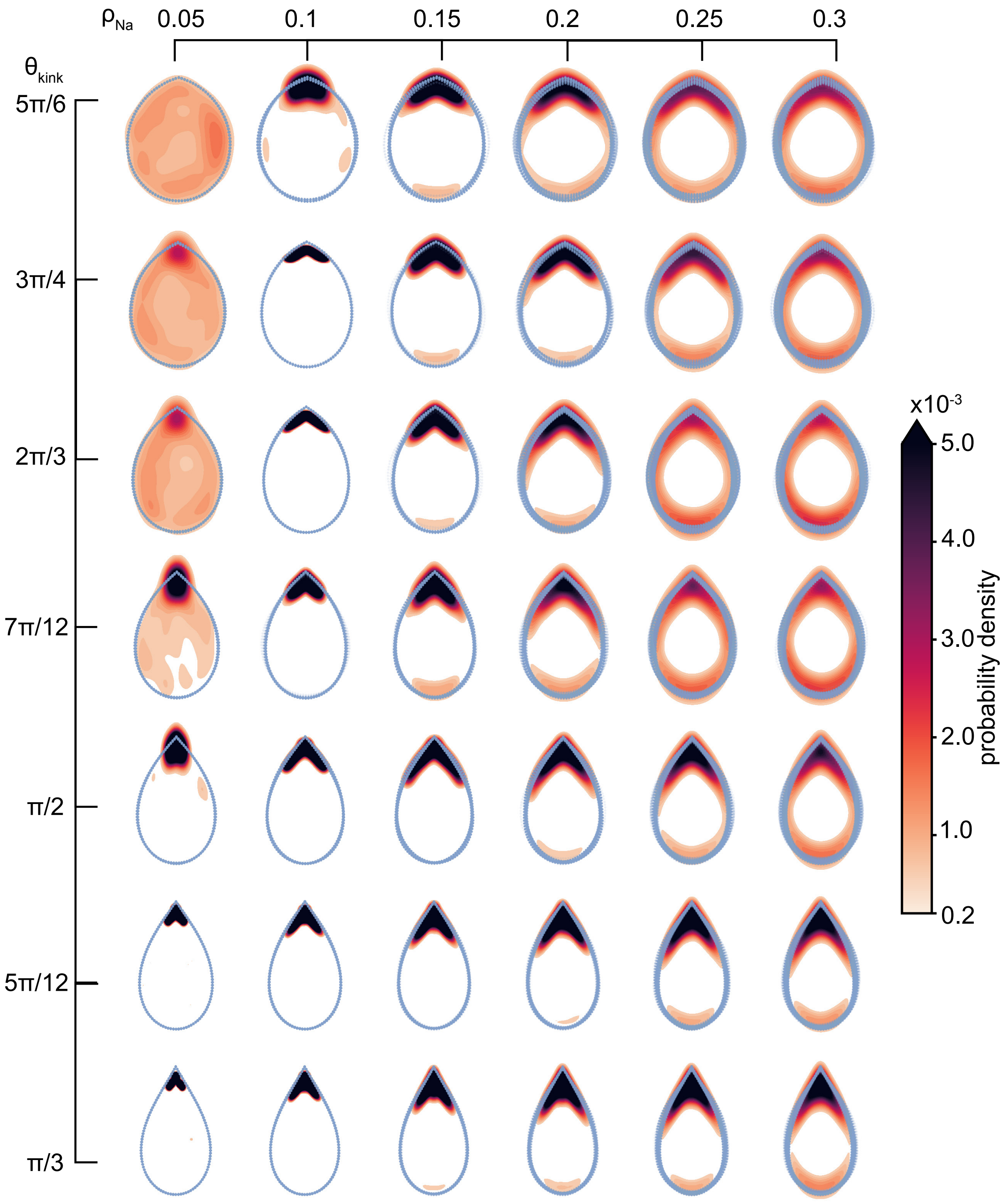

Figure S10. Probability density plot of enclosed particle $\alpha = 3$, $Pe = 50$, $\kappa_a = 1000 k_B T$. The color scheme is the same as in Fig. 2

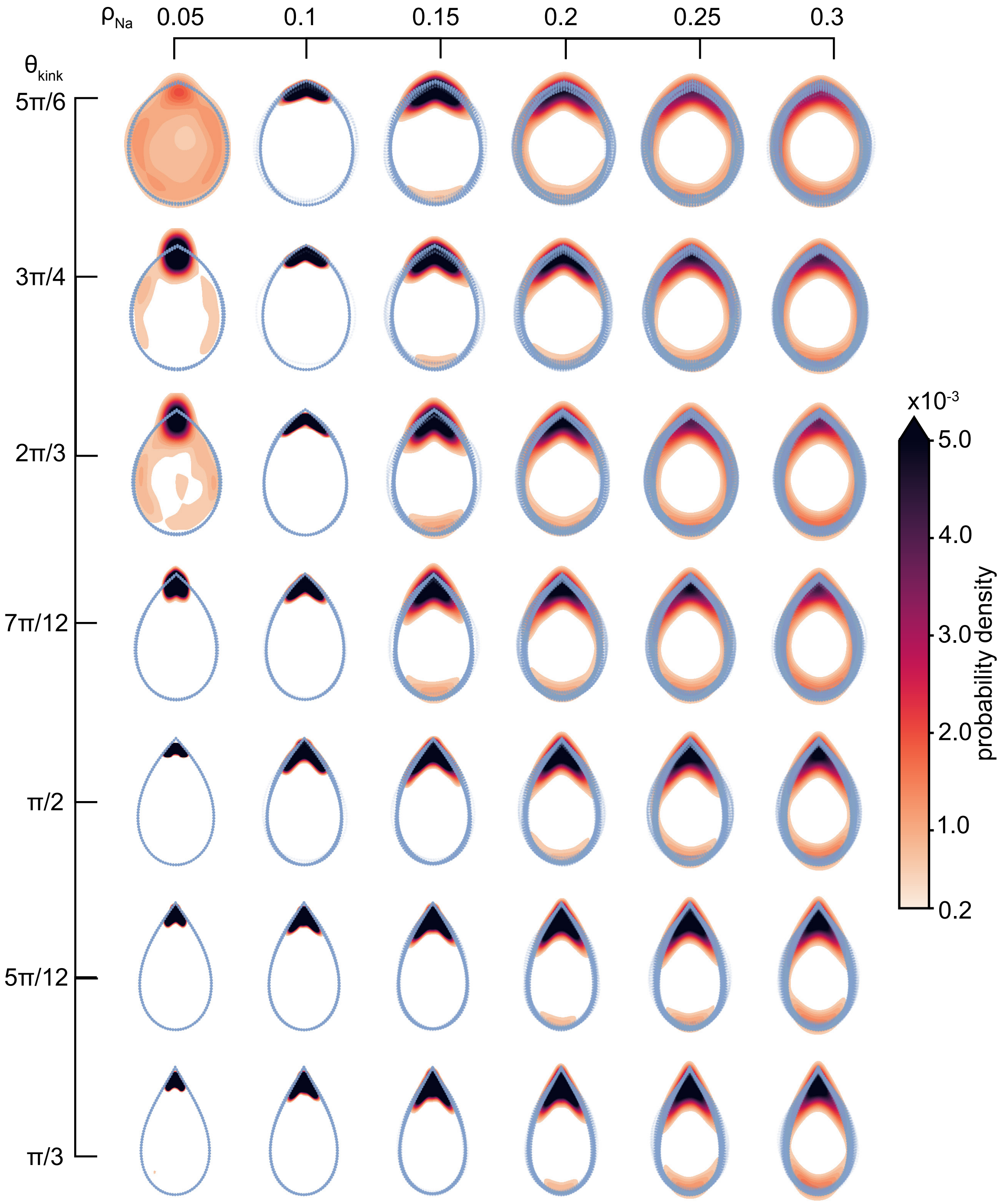

Figure S11. Probability density plot of enclosed particle $\alpha = 3$, $Pe = 75$, $\kappa_a = 1000 k_B T$. The color scheme is the same as in Fig. 2

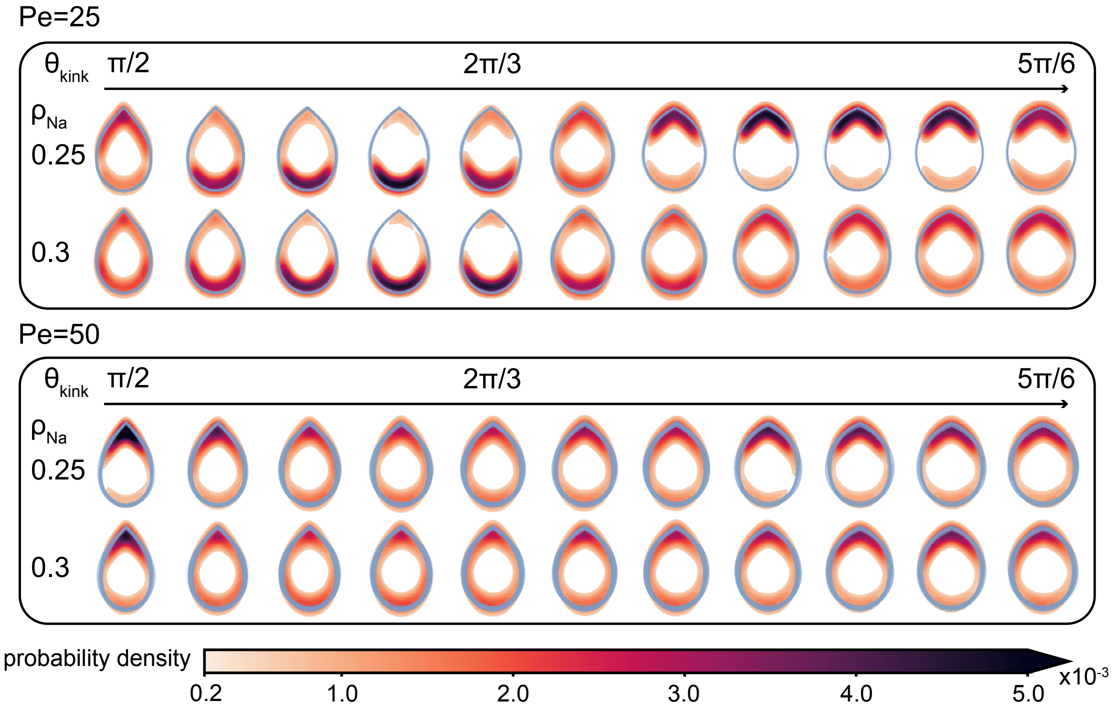

Figure S12. Flipped trend in probability density plot of enclosed particle with various θ_{kink} $\alpha = 3$, $Pe = 25, 50$, $\kappa_a = 1000k_BT$. The color scheme is the same as in Fig. 2

References

1. Xin, W., Wu, H., Grason, G. M. & Santore, M. M. Switchable positioning of plate-like inclusions in lipid membranes: Elastically mediated interactions of planar colloids in 2D fluids. *Sci. Adv.* **7**, eabf1943, DOI: 10.1126/sciadv.abf1943 (2021).
